# Supplementary material for: The psychosis risk factor RBM12 encodes a novel repressor of GPCR/cAMP signal transduction
Source: J Biol Chem. 2023 Aug 4;299(9):105133. doi: 10.1016/j.jbc.2023.105133 (PMC10502367; doi:10.1016/j.jbc.2023.105133)
Supplement: Table S3 [file mmc3.docx]

**Table S4. CRISPR KO and CRISPRi gRNA sequences used in this paper.**

| Name | Guide Sequence | Forward Primer  (5’ - 3’) | Reverse Primer  (5’ - 3’) |
| --- | --- | --- | --- |
| *RBM12* CRISPR KO gRNA #1 | AAGGTCAAGATCACCACATG | CACCGAAGGTCAAGATCACCACATG | AAACCATGTGGTGATCTTGACCTTC |
| *RBM12* CRISPR KO gRNA #2 | AAATGATACTAAATCCAGAG | CACCGAAATGATACTAAATCCAGAG | AAACCTCTGGATTTAGTATCATTTC |
| NTC CRISPRi gRNA | GGCAGGGCGTGGCGGGCGGTA | TTGGGCAGGGCGTGGCGGGCGGTAGTTTAAGAGC | TTAGCTCTTAAACTACCGCCCGCCACGCCCTGCCCAACAAG |
| *RBM12* CRISPRi gRNA | GAGGAGGTGGTGGCTGCGTT | TTGGAGGAGGTGGTGGCTGCGTTGTTTAAGAGC | TTAGCTCTTAAACAACGCAGCCACCACCTCCTCCAACAAG |
