# Supplementary material for: The psychosis risk factor RBM12 encodes a novel repressor of GPCR/cAMP signal transduction
Source: J Biol Chem. 2023 Aug 4;299(9):105133. doi: 10.1016/j.jbc.2023.105133 (PMC10502367; doi:10.1016/j.jbc.2023.105133)
Supplement: Table S4 [file mmc4.docx]

**Table S5. RT-qPCR primers used in this paper**

| Target Gene  (*Homo sapiens*) | Forward Primer (5’-3’) | Reverse Primer (5’-3’) |
| --- | --- | --- |
| *GAPDH* | CAATGACCCCTTCATTGACC | GACAAGCTTCCCGTTCTCAG |
| *PCK1* | CTGCCCAAGATCTTCCATGT | CAGCACCCTGGAGTTCTCTC |
| *NR4A1* | AGTGCAGAAAAACGCCAAGT | TTCGGACAACTTCCTTCACC |
| *FOS* | GCCTCTCTTACTACCACTCACC | AGATGGCAGTGACCGTGGGAAT |
| *RBM12* | GCCAAAGTCTGTGCCCACATAAC | GAACCAATGCCTGTCCTAGACC |
| *ADRB2* | GATTTCAGGATTGCCTTCCA | TATCCACTCTGCTCCCCTGT |
