# Supplementary material for: The psychosis risk factor RBM12 encodes a novel repressor of GPCR/cAMP signal transduction
Source: J Biol Chem. 2023 Aug 4;299(9):105133. doi: 10.1016/j.jbc.2023.105133 (PMC10502367; doi:10.1016/j.jbc.2023.105133)
Supplement: Supporting information [file mmc5.pdf]

Supplementary Data

The psychosis risk factor *RBM12* encodes a novel repressor of GPCR/cAMP signal transduction

Khairunnisa M. Semesta[1*], Angelica Garces[1*] and Nikoleta G. Tsvetanova[1#]

[1]Department of Pharmacology and Cancer Biology, Duke University, Durham, NC, United States

*equal contribution, [#]corresponding author: nikoleta.tsvetanova@duke.edu

Supplementary Figures

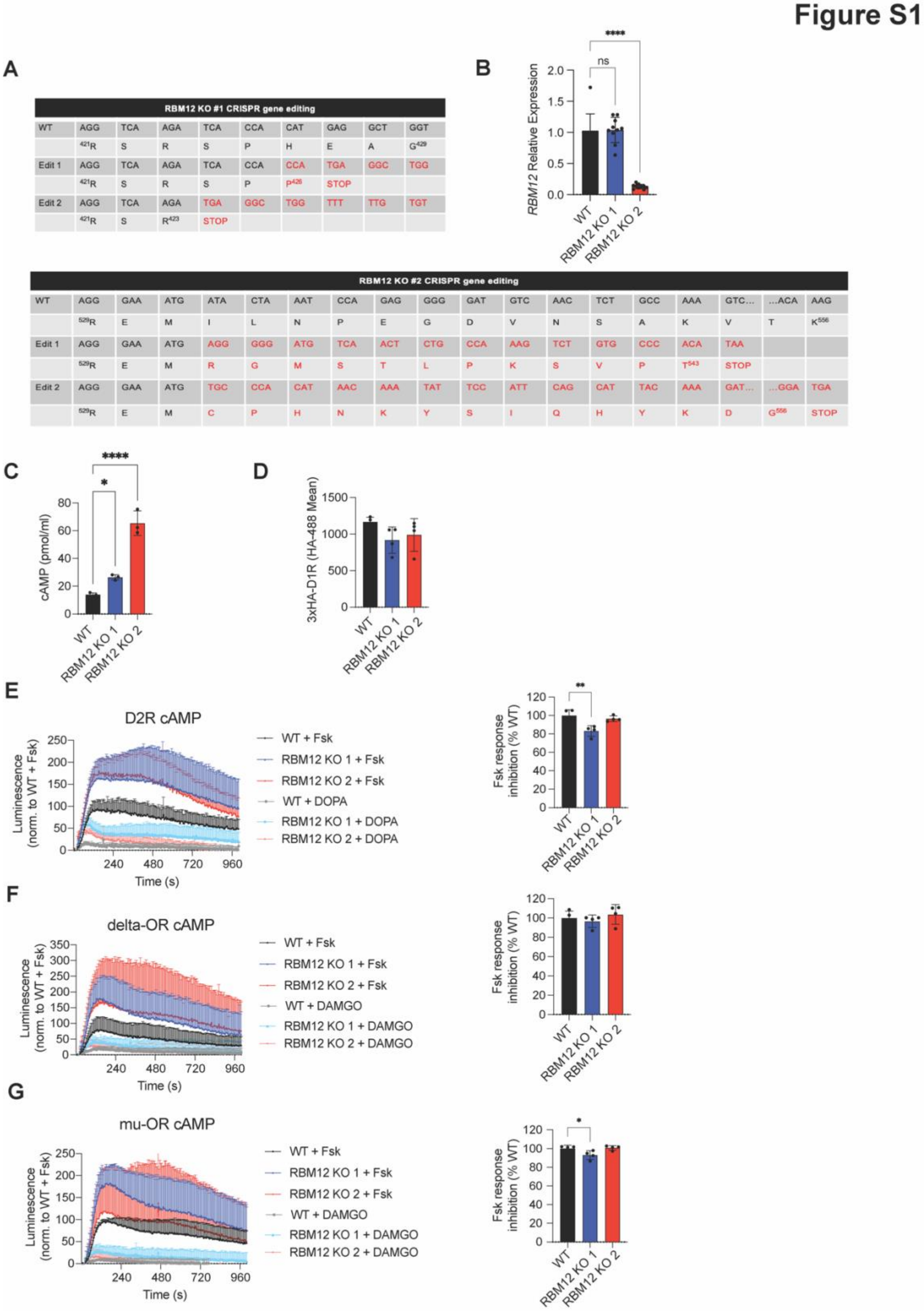

RBM12 KO #1 CRISPR gene editing									
WT	AGG	TCA	AGA	TCA	CCA	CAT	GAG	GCT	GGT
	^{421}R	S	R	S	P	H	E	A	G^{429}
Edit 1	AGG	TCA	AGA	TCA	CCA	CCA	TGA	GGC	TGG
	^{421}R	S	R	S	P	P^{426}	STOP		
Edit 2	AGG	TCA	AGA	TGA	GGC	TGG	TTT	TTG	TGT
	^{421}R	S	R^{423}	STOP					

RBM12 KO #2 CRISPR gene editing																		
WT	AGG	GAA	ATG	ATA	CTA	AAT	CCA	GAG	GGG	GAT	GTC	AAC	TCT	GCC	AAA	GTC...	...ACA	AAG
	^{529}R	E	M	I	L	N	P	E	G	D	V	N	S	A	K	V	T	K^{556}
Edit 1	AGG	GAA	ATG	AGG	GGG	ATG	TCA	ACT	CTG	CCA	AAG	TCT	GTG	CCC	ACA	TAA		
	^{529}R	E	M	R	G	M	S	T	L	P	K	S	V	P	T^{543}	STOP		
Edit 2	AGG	GAA	ATG	TGC	CCA	CAT	AAC	AAA	TAT	TCC	ATT	CAG	CAT	TAC	AAA	GAT...	...GGA	TGA
	^{529}R	E	M	C	P	H	N	K	Y	S	I	Q	H	Y	K	D	G^{556}	STOP

Supplementary Figure 1. Characterization of RBM12 KO cell lines. (A) Sanger sequencing of PCR-amplified genomic DNA from wild-type and RBM12 knockout clones showing positions of indels in each allele. (B) RT-qPCR of *RBM12* mRNA expression in the knockouts (n = 10). (C) cAMP levels in wild-type and HEK293 RBM12 knockouts measured using ELISA assay (n = 3). Cells were treated with 1 μM isoproterenol for 5 min. All quantities were normalized to total protein concentration per sample. (D) Flow cytometry analysis showing comparable 3xHA-D1R expression in WT or RBM12 knockout cells transfected with plasmid encoding the receptor and surface-labeled with anti-HA-488 antibody (n = 4). (E-G) Luminescent GloSensor measurement of cAMP accumulation in cells overexpressing D2R (F, n = 4), ΔOR (G, n = 4), and μOR (H, n = 4) in response to either 10 μM forskolin and vehicle (DMSO) or 10 μM forskolin and 10 μM DOPA (D2R) in the presence of ICI-118,551 to isolate the D2R response (D), or 10 μM DAMGO (ΔOR and μOR) (E-F). All data are mean ± SD. Statistical significance was determined using one-way ANOVA with Dunnett's correction (B, C, E-G). *** = $p < 0.001$, ** = $p < 0.01$, * = $p < 0.05$

Figure S2

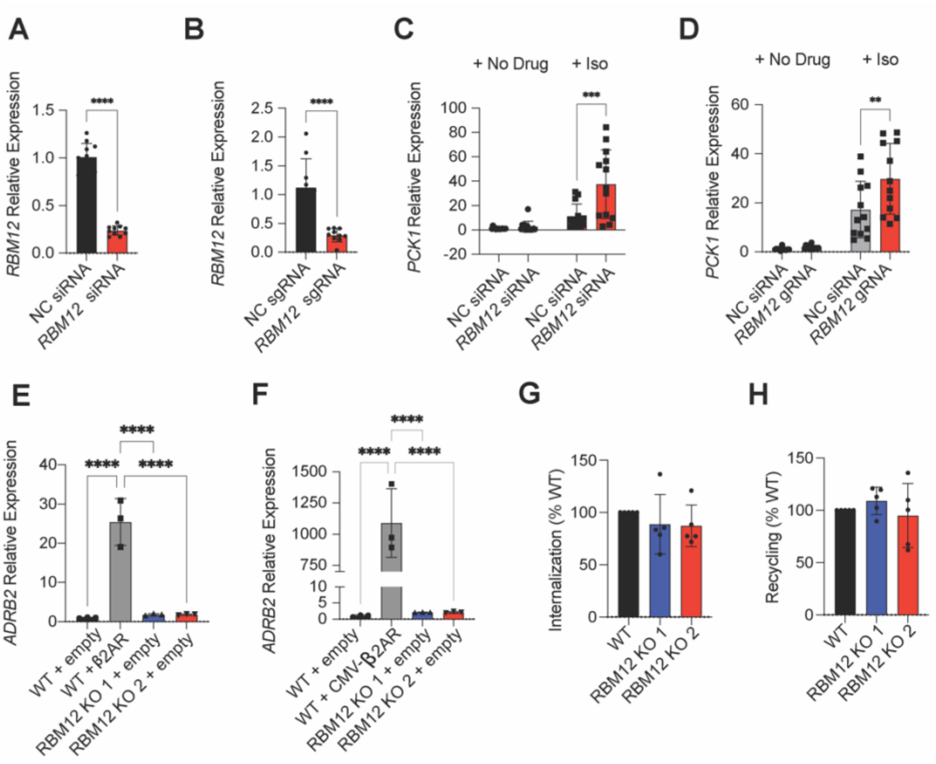

Supplementary Figure 2. Knockdown efficiencies of different strategies to deplete RBM12 and characterization of β2AR overexpression. (A-B) *RBM12* expression in cells transfected with non-targeting control (NC)- or *RBM12*-siRNA (A, n = 11), or NTC or *RBM12* CRISPRi gRNA (B, n = 10). (C) *PCK1* expression in cells transfected with non-targeting WT or *RBM12*-targeting siRNA, untreated or treated with 1 μM Iso for 1 hour (n = 13-14). (D) *PCK1* expression by RT-qPCR in cells expressing NTC or *RBM12*-targeting CRISPRi gRNA, untreated or treated with 1 μM Iso for 1 hour (n = 12). (E) *ADRB2* expression in cells transfected with empty plasmid or plasmid construct expressing β2AR from endogenous promoter (n = 3). (F) *ADRB2* expression in cells transfected with empty plasmid or plasmid construct expressing β2AR under a CMV promoter (n = 3). (G-H) Flow cytometry analysis of FLAG-β2AR internalization (G, n = 5) and recycling (H, n = 5). All data are mean ± SD. Statistical significance was determined using unpaired two-sided Student *t*-test (A-B), two-way ANOVA with Tukey's correction (C-D), or one-way ANOVA with Tukey's correction. **** = $p < 0.0001$, *** = $p < 0.001$, ** = $p < 0.01$

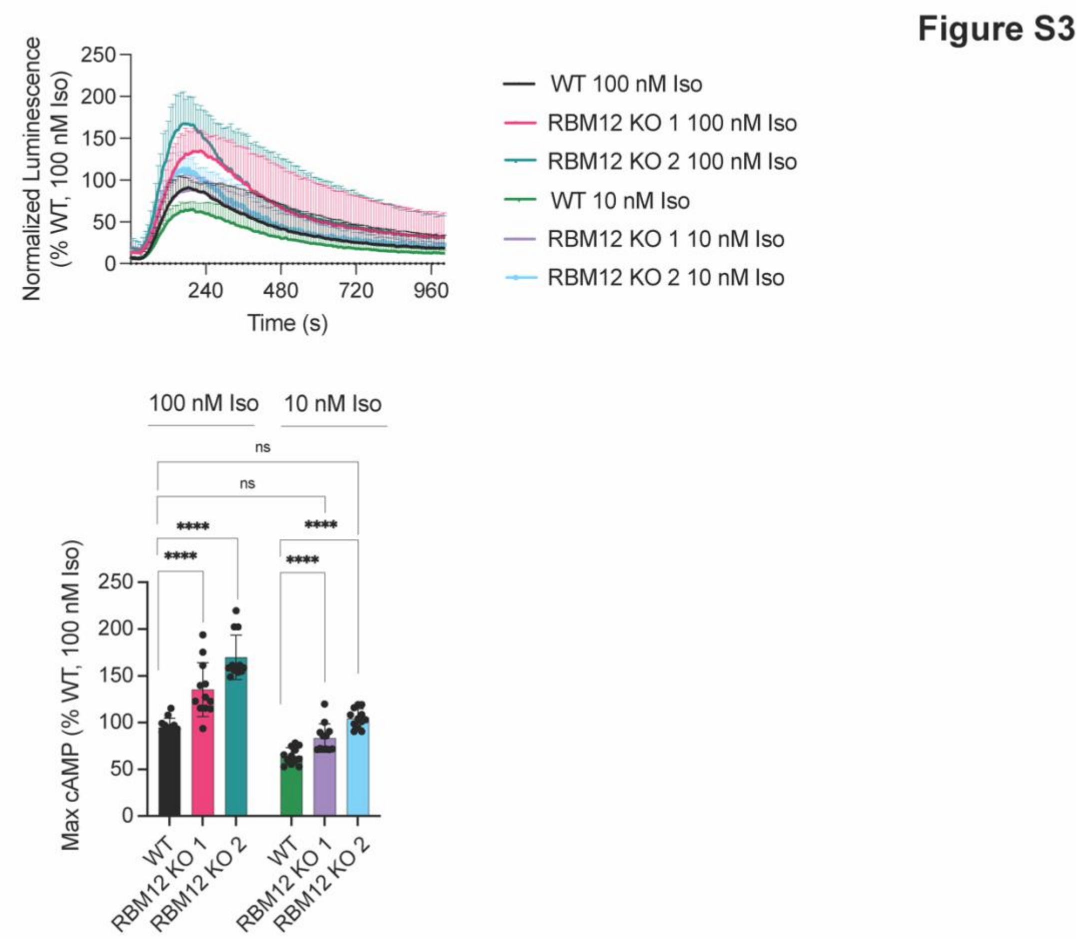

Supplementary Figure 3. Isoproterenol dose-response measurement of cAMP production in wild-type and RBM12 KO cells. Luminescent GloSensor measurement of cAMP accumulation following treatment with either 100 μM isoproterenol in wild-type cells or 10 nM isoproterenol in RBM12 knockout cells (n = 12). All data are mean ± SD. Statistical significance was determined using two-way ANOVA with Tukey's correction. **** = $p < 0.0001$

Figure S4

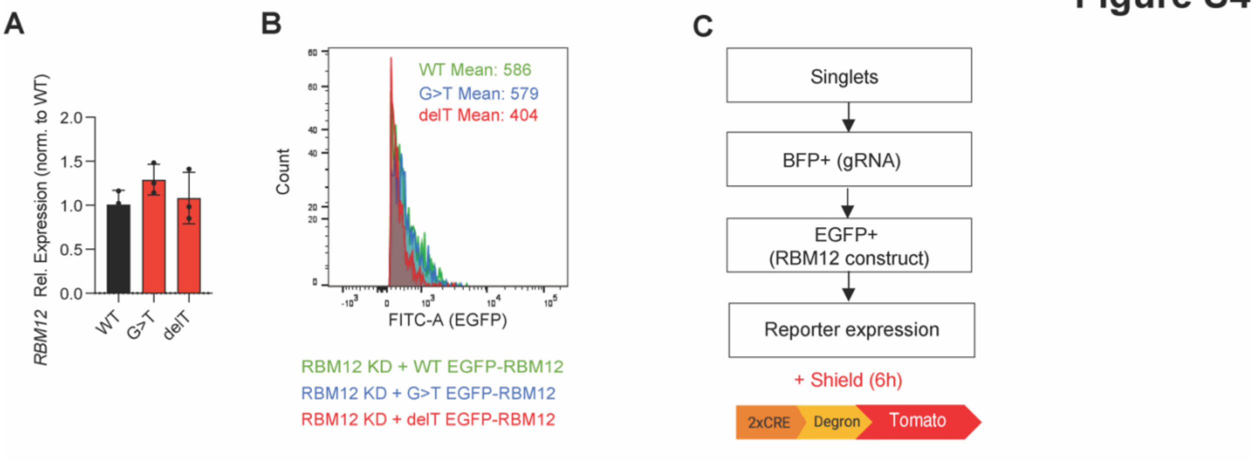

Supplementary Figure 4. Wild-type and mutant RBM12 expression in HEK293 cells. (A) Expression of *RBM12* mRNA by RT-qPCR in cells transfected with plasmid encoding EGFP-tagged WT, G>T, or delT RBM12 (n = 3). All data are mean ± SD. (B) Flow cytometry measurement of WT, G>T, or delT EGFP-RBM12 expression in the rescue assay. (C) Schematic of flow cytometry analysis strategy in the rescue assay.

Figure S5

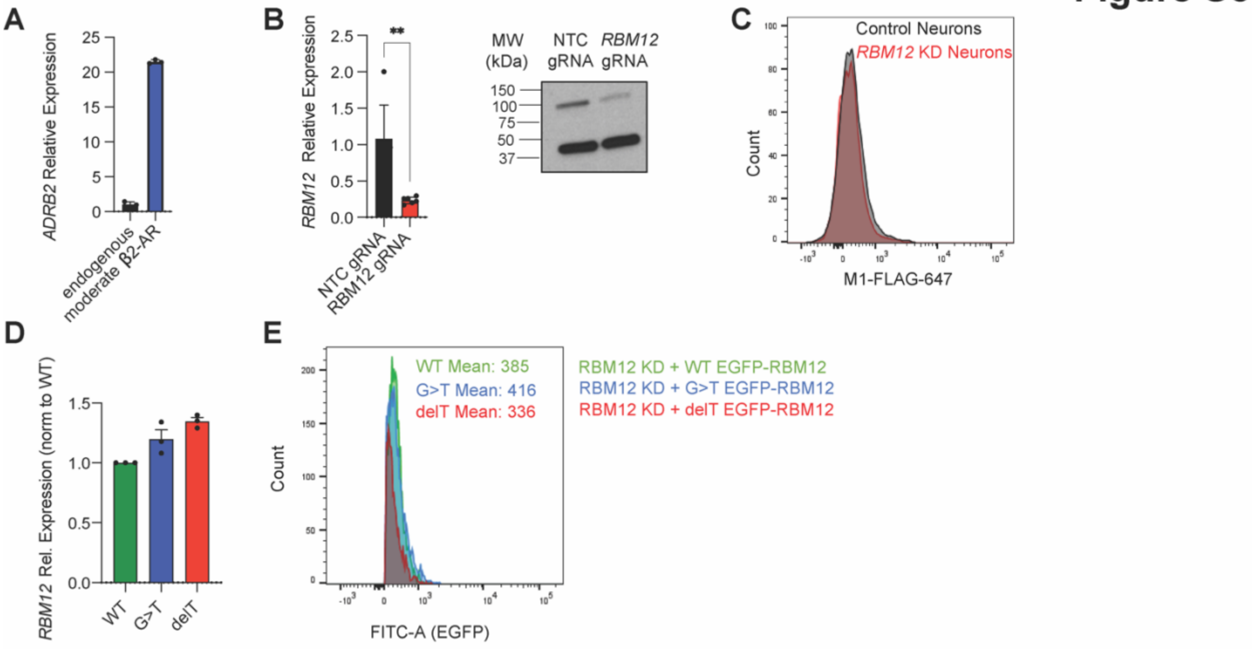

Supplementary Figure 5. Generation and characterization of RBM12-depleted human neurons. (A) *ADRB2* mRNA expression (n = 3) in neurons derived from the parental hiPSC cell line expressing native receptors versus neurons derived from hiPSC cell line overexpressing the β2-AR. (B) *RBM12* expression (n = 6) and representative Western blot showing CRISPRi-dependent *RBM12* depletion in iNeurons. See also Figure S7. (C) Flow cytometry analysis of FLAG-tagged β2-AR expression in wild-type and *RBM12* KD neurons. (D) *RBM12* mRNA levels in neurons expressing WT, G>T, or delT EGFP-RBM12 in the rescue assay (n = 3). (E) Flow cytometry analysis of vector, WT, G>T, or delT EGFP-RBM12 expression in the neuron rescue assay. All data are mean ± SD. Statistical significance was determined using unpaired two-tailed Student *t*-test (B). ** = $p < 0.01$

Figure S6

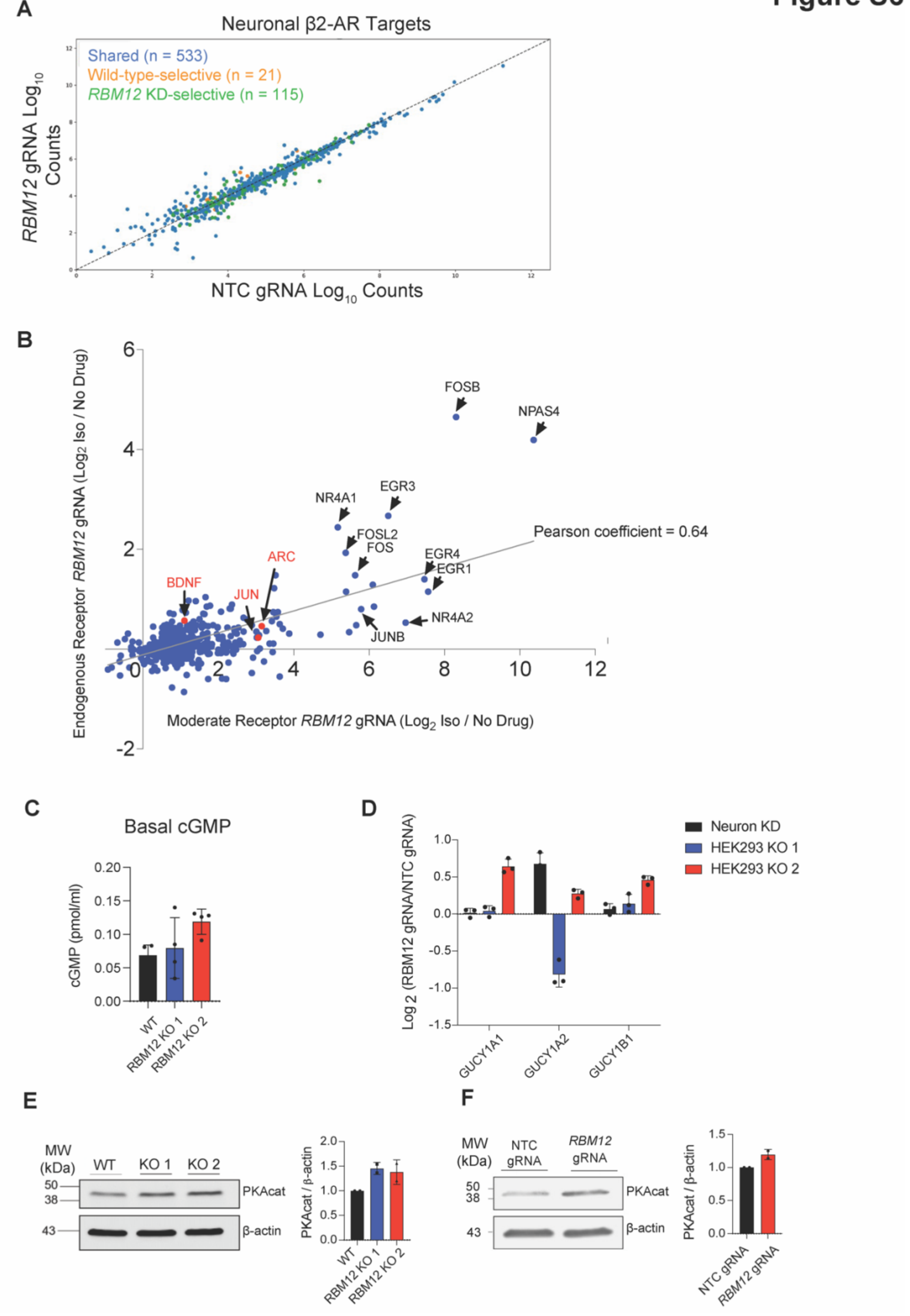
A
Neuronal β2-AR Targets
Shared (n = 533)
Wild-type-selective (n = 21)
RBM12 KD-selective (n = 115)
RBM12 gRNA Log10 Counts
NTC gRNA Log10 Counts
B
FOSB
NPAS4
NR4A1
EGR3
FOSL2
FOS
EGR4
EGR1
NR4A2
JUNB
ARC
BDNF
JUN
Pearson coefficient = 0.64
Endogenous Receptor RBM12 gRNA (Log2 Iso / No Drug)
Moderate Receptor RBM12 gRNA (Log2 Iso / No Drug)
C
Basal cGMP
cGMP (pmol/ml)
WT
RBM12 KO 1
RBM12 KO 2
D
Log 2 (RBM12 gRNA/NTC gRNA)
Neuron KD
HEK293 KO 1
HEK293 KO 2
GUCY1A1
GUCY1A2
GUCY1B1
E
MW (kDa)
WT
KO 1
KO 2
PKAcat
β-actin
PKAcat / β-actin
WT
RBM12 KO 1
RBM12 KO 2
F
MW (kDa)
NTC gRNA
RBM12 gRNA
PKAcat
β-actin
PKAcat / β-actin
NTC gRNA
RBM12 gRNA

Supplementary Figure 6. Basal expression of neuronal β2-AR target genes is unaffected by RBM12 depletion. (A) Scatter plot of normalized RNAseq counts of neuronal β2-AR-dependent transcriptional targets in untreated cells (n = 669 genes). Blue dots represent genes that were induced by 1 hour 1 μM Iso treatment in both wild-type and *RBM12* KD neurons. Orange dots represent genes that were induced only in wild-type and unchanged or downregulated in *RBM12* KD neurons. Green dots represent genes that were induced only in *RBM1*2 KD neurons and unchanged or downregulated in wild-type. Indicated by arrows are a subset of genes with established roles in neuronal activity. The underlying information is summarized in Table S1. (B) Linear regression analysis between isoproterenol-induced abundance changes in transcriptional targets in RBM12-depleted neurons from the parental iPSC line versus neurons derived from iPSC moderately overexpressing the receptor. (C) Basal cGMP levels in wild-type and HEK293 RBM12 knockouts measured using ELISA assay (n = 4). (D) mRNA expression changes (Log_2 RBM12-depleted/wild-type) for the three soluble guanylyl cyclase isoforms from RNAseq analysis of neurons or HEK293 cells depleted of RBM12. No statistically significant differences trending in the same direction across the three cell lines were found by Wald test. (E) Representative Western blot and quantification of the catalytic subunit of PKA (PKAcat) in wild-type and RBM12 knockout HEK293 cells, normalized to wild-type values per experiment (n = 2). See also Figure S7. (F) Representative Western blot and quantification of the catalytic subunit of PKA (PKAcat) in wild-type and *RBM12* knockdown neurons, normalized to wild-type values per experiment (n = 2). See also Figure S7.

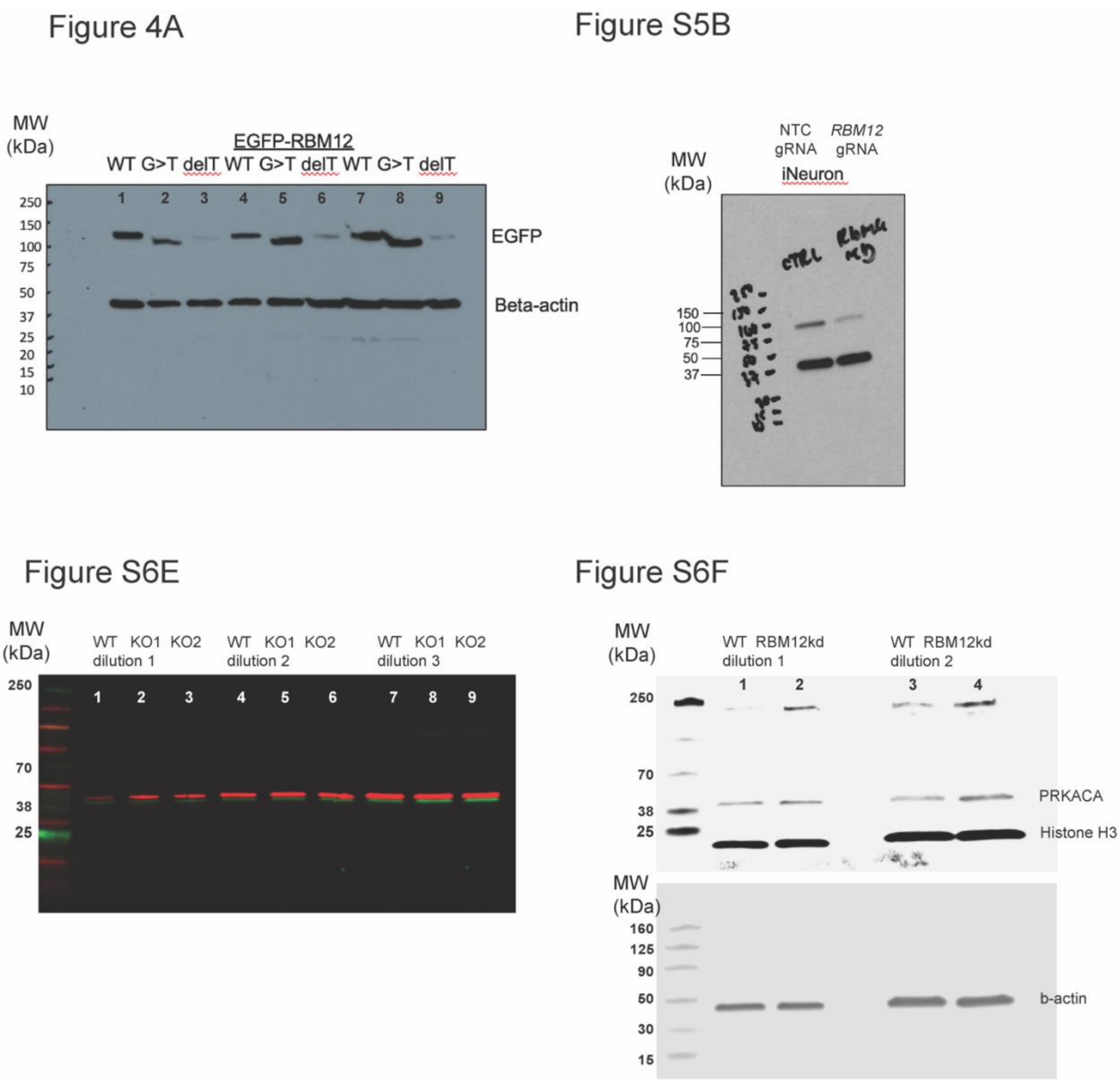

Supplementary Figure 7. Uncropped Western blots. Figure 4a displays lanes 4-6 of the uncropped blot. Figure S6e: anti-beta-actin is in red, anti-PRKACA is in green. Figure S6 displays lanes 7-9. Figure S6f displays anti-PRKACA and anti-beta actin from lanes 3-4.

Supplementary Tables

Table S1. Neuronal β2-AR transcriptional target genes identified by RNA-seq.

Table S2. Expression of wild-type and *RBM12* knockdown neurons and Gene Ontology analysis.

Table S3. CRISPR KO and CRISPRi gRNA sequences used in this paper.

Table S4. RT-qPCR primers used in this paper
